# Willingness of female sex workers to use HIV self-testing in Burkina Faso: analysis of cross-sectional respondent-driven sampling data

**DOI:** 10.3389/fpubh.2025.1528270

**Published:** 2025-04-11

**Authors:** Kadari Cisse, Henri Gautier Ouedraogo, Odette Ky-Zerbo, Dinanibè Kambire, Désiré Lucien Dahourou, Ter Tiero Elias Dah, Sylvie Zida, Célestine Ki-Toe, Solange Dioma, Fatou Sissoko, Alexandre Yugbare, Abdoul Ibrahim Ouedraogo, Smaila Ouedraogo, Seni Kouanda

**Affiliations:** ^1^Institut de Recherche en Sciences de la Santé (IRSS), Centre National de Recherche Scientifiques de Technologique (CNRST), Ouagadougou, Burkina Faso; ^2^Unité de Formation et de Recherche en Sciences de la Santé (UFR SS), Université Ledéa Bernard Ouédraogo, Ouahigouya, Burkina Faso; ^3^Sécrétariat Permanent du Conseil National de Lutte contre le SIDA et les IST (SP/CNLS-IST), Ouagadougou, Burkina Faso; ^4^Unité de Formation et de Recherche Sciences de la Santé (UFR/SDS), Université Joseph Ki-Zerbo, Ouagadougou, Burkina Faso; ^5^Institut Africain de Santé Publique, Ouagadougou, Burkina Faso

**Keywords:** HIV self-testing, female sex worker, willingness, RDS, Burkina Faso

## Abstract

**Background:**

HIV self-testing (HIVST) is an approach that allows individuals to test for HIV and obtain results in their homes or other private settings. It is recommended by the World Health Organization (WHO), particularly for key populations, to help achieve the first 95. The objective of this study was to evaluate the willingness to use HIVST among female sex workers (FSWs) in Burkina Faso.

**Methods:**

We performed a biobehavioral cross-sectional study conducted in five regions in Burkina Faso. FSWs were recruited using the respondent-driven sampling (RDS) method. To evaluate FSW’s willingness to use HIVST, each participant was invited to answer a “Yes” or “No” question: “If you were offered an HIV self-test, would you use it?.” The determinants of willingness to use HIVST were identified using Poisson-modified generalized estimating equation models.

**Results:**

A total of 1,338 FSWs were included in this study. The mean age was 28.1 years (standard deviation: 7.71 years), and 47.9% of FSWs had attended at least secondary school. Nearly 21.1% had good knowledge of HIV transmission modes. One-third (28.8%) of FSWs reported not using a condom during their last sexual intercourse with a non-paying partner. Among the study participants, 89.5% (95% CI: 87.0; 91.6) were willing to use HIVST. The main determinants of FSW’s willingness to use HIVST were being married (adjusted prevalence ratio (aPR): 1.10 (95%CI: 1.01; 1.20) *p* = 0.034), having first sex at the age greater than 18 years (aPR: 1.14 (95%CI: 1.02; 1.29) *p* = 0.024), being member of an association (aPR: 1.10 (95%CI: 1.02; 1.18) *p* = 0.014), and being no current alcohol consumer (aPR: 1.06 (95%CI 1.01; 1.12) *p* = 0.026).

**Conclusion:**

This study revealed a good willingness to use HIVST among FSWs. This is a promising means to increase HIV testing coverage and knowledge of HIV status among FSWs and would then strengthen HIV prevention and care services.

## 1 Introduction

Efforts have significantly reduced the HIV burden worldwide. However, it remains a major public health issue. Indeed, by the end of 2022, the number of people living with HIV (PLHIV) worldwide was estimated at 39.0 million, including 1.3 million new infections (1). In 2022, female sex workers accounted for 7.7% of new infections worldwide, whereas in West and Central Africa, they accounted for 15% (2).

Burkina Faso has a concentrated HIV epidemic, with an HIV prevalence of less than 1% in the general population and a higher prevalence among vulnerable and key populations (i.e., female sex workers (FSWs) and men who have sex with men). The prevalence of HIV among FSWs was 10.3% (3) in 2016 and ranged from 13 to 30.1% in 2017 according to location (4). Like many other SSA countries, Burkina Faso has subscribed to the UNAIDS target of 95–95-95, which suggests that by 2025, 95% of people living with HIV are aware of their status, 95% of those who are aware of their status receive antiretroviral therapies (ART), and 95% of those who receive ART have a suppressed viral load (5). Few studies have assessed the progress to this target (5). However, about the first 95, only 83% of PLVIH knew their status in 2023 in Burkina Faso (6). To achieve the first goal, new approaches such as index testing and HIV self-testing (HIVST) have been proposed to reach most at-risk populations, who are usually less likely to use health facility-based testing (7).

HIVST can help eliminate some obstacles regarding HIV testing, such as the stigmatization faced by key populations, particularly FSWs in health facilities, and transportation fees. HIVST is a procedure whereby a person uses a simple oral swab or blood sample to perform an HIV test, which he or she will interpret alone, often in a private place. This is often done using rapid test. When the test is positive, the result needs to be confirmed by a health professional using national algorithm. In terms of approach, HIVST represents an innovation with the goal of increasing the uptake of testing among the hidden and/or the most at-risk populations, including FSW. Most studies have shown that the majority of individuals tested prefer the testing process to be discreet and confidential, especially for populations that are victims of stigmatization, such as FSW. This might overcome the lower rate (close to 57.8%) of HIV testing reported in a previous study conducted in Burkina Faso (8). Since 2016, the World Health Organization has recommended that HIVST be offered as an additional HIV testing strategy in sub-Saharan Africa (7). However, HIVST has not been sufficiently implemented. A recent study revealed a lower rate of HIV testing among FSWs in Burkina Faso (8). The government has adopted a national strategy for 2021–2025, which aims to reduce new HIV infections by 75% and improve HIV testing among FSWs (9). Despite the ongoing implementation of new national strategy, little is known about HIVST among the key population in Burkina Faso. This study aimed to investigate the willingness to use HIVST and its determinants among FSWs in Burkina Faso.

## 2 Materials and methods

### 2.1 Study sites

The study was conducted in the five main large cities of Burkina Faso (Ouagadougou, Bobo-Dioulasso, Koudougou, Tenkodogo, and Ouahigouya). Due to the specificity of the hidden populations, these cities were selected according to several criteria: geographical disparities in HIV prevalence, cultural variation, and crowds of sexual activities in these cities.

### 2.2 Study type and period

A cross-sectional survey was carried out via the respondent-driven sampling method, which is one of the best methods to reach hidden populations, such as FSW (10, 11). The survey took place from June to August 2022.

### 2.3 Study population

The study population included female sex workers (FSWs) living in the five study towns. Female sex workers are defined as “girls and women over the age of 18 who receive money or other gifts in exchange for sexual services, either regularly or occasionally.” The inclusion criteria were as follows: being female, at least 15 years old, having sexual intercourse in exchange for money as the main income source for 12 months, having a valid coupon (the validity of the coupon was set at 15 days), and consenting to participate in the study. HIV-positive FSWs were excluded from this analysis. We included FSWs with less than 18 years old because a previous study showed an important proportion of them who started sex work as minors (12).

### 2.4 Sample size and sampling

The sampling process and sample size calculation have been described elsewhere (8). In brief, the sample size was 1,424 FSWs to be recruited. The sample size was calculated using the Salganik (13) formula. The sampling followed the RDS methods. The recruitment started with two seeds in each city. Seeds were FSWs known as the leader of their community with a high network size. The seeds who meet the study eligibility were non-randomly selected with the contributions of NGOs and FSW associations. They were selected considering their influence on the FSW community. They are known as leaders in the FSW community of their city. After their inclusion in the study, they were trained on the process of recruitment before receiving three coupons to recruit other FSWs. Second, the following participant also received three coupons to recruit other participants until the sample size was reached. Each coupon was identified by unique numbers; thus, each person recruited can be identified by his coupon number. This allows us to know the order of recruitment and reduce any risk of double inclusion in the survey.

### 2.5 Data collection process

The study methods have been described elsewhere (8). In brief, following the RDS approach, data collection started by checking the eligibility of the participants. If a participant was eligible, he was invited to complete a face-to-face questionnaire administered by a trained investigator. The questionnaire was structured to include sociodemographic and behavioral data and was programmed on a digital tablet via the CSPro application. After receiving an explanation of what HIVST consists of, participants were invited to answer the following question: “If you were offered an HIV self-test, would you use it?” After the interviews, the participants underwent HIV testing via a rapid diagnosis test (RDT).

### 2.6 Study variables

The outcome variable in our study was the willingness to use the HIVST kit. The outcome variable was treated as a binary variable: FSWs who agreed with self-testing (code = 1) versus those who did not agree (code = 0).

The independent variables include demographic and socioeconomic variables, behavioral factors, and HIV knowledge variables. These variables were age (<25 years and >=25 years) of FSW, current marital status, educational level, monthly income (Dollar), age at first sex (<18 and >=18), age at initiation of sex work (<18 and >=18), history of drug use (Yes/No), condom use at last sex with clients (Yes/No), condom use at last sex with partners (Yes/No), history of previous incarceration in last 12 months (Yes/No), current alcohol consumption (Yes/No) with refer to alcohol consumption within last 30 days, lifetime history of drug use (Yes/No), HIV knowledge (Sufficient/Non-sufficient) as defined by UNAIDS, number of biological children (neither, one, two or three, more than three), and membership in an association (Yes/No) also known as peer group membership (it seems to be a way for HIV prevention among female sex workers including HIVST delivery (14, 15)).

### 2.7 Data processing and analysis

After the data were cleaned, we first excluded all participants who tested positive for HIV. We then describe the characteristics of the participants. We estimated the rate of FSW willingness to use HIVST via the unweighted and RDS weighted options. For each rate, a confidence interval was calculated. The rate of willingness to use HIVST was presented by the demographic and risk behavior characteristics of the participants. We conducted a modified Poisson regression using a generalized estimating equation with an exchangeable correlation structure as recommended (16) to identify factors associated with willingness to use HIVST among female sex workers. From this model, we derive the prevalence ratio and its confidence intervals. All analyses were performed using Stata 18. All the statistical tests were considered significant when the *p*-value was <5%.

### 2.8 Ethical consideration

The protocol of this study was approved by the National Ethical Committee for Health Research (Deliberation Number 2020-02-029 of 5 February 2020).

## 3 Results

### 3.1 Participant characteristics

Among the 1,425 participants, 87 HIV-positive individuals were excluded. The final sample size for this analysis was 1,338 FSWs. The participants’ characteristics are presented in Table 1.

**Table 1:** Willingness to use HIV self-testing among FSWs by different subgroups of demographics and risk behaviors in Burkina Faso.

Variable	Total	Number of FSWs willing for HIV self-test	Unweighted prevalence (95%CI)	RDS weighted prevalence (95%CI)
Overall	1,338	1,082	89.4 (87.6; 91.0)	89.5 (87.0; 91.6)
Age (year)				
<25	529	408	87.7 (84.4; 90.4)	88.8 (84.4; 92.1)
> = 25	809	674	90.6 (88.3; 92.5)	89.9 (86.8; 92.4)
Current marital status				
Single	970	788	90.0 (87.8; 91.8)	90.2 (87.3; 92.5)
Married	30	25	96.2 (74.6; 99.5)	93.9 (61.6;99.3)
Divorced or widowed	338	269	87.6 (83.4; 90.9)	87.2 (81.4; 91.3)
Educational level				
Illiterate	310	245	88.4 (84.1; 91.7)	89.2 (83.5; 93.0)
Primary school	350	292	90.7 (87.0; 93.4)	85.8 (79.6; 90.3)
Secondary or high school	678	545	89.3 (86.6; 91.6)	91.8 (88.6; 94.1)
Monthly income (dollars)				
14$–100$	110	85	90.4 (82.4; 95.0)	87.2 (72.5; 94.7)
100$–250$	528	427	91.0 (88.1; 93.3)	89.8 (85.5; 92.9)
250$–700$	564	461	89.2 (86.2; 91.6)	90.6 (87.0; 93.3)
700$–5014$	136	109	84.5 (77.1; 89.8)	85.8 (76.4; 91.8)
Age at first sex				
<18	744	608	90.7 (88.3; 92.7)	90.7 (87.3; 93.3)
> = 18	594	474	87.9 (84.9; 90.4)	88.0 (84.1; 91.1)
Age at initiation of sex work				
<18	181	138	86.8 (80.5; 91.3)	85.1 (76.2;91.0)
> = 18	1,157	944	89.9 (87.9; 91.6)	90.4 (87.8; 92.4)
History of injection drug use (ever)				
Yes	11	10		
No	1,324	1,072	89.4 (87.5; 91.0)	89.4 (86.9; 91.5)
Condom use at last sex with paying partner				
Yes	1,130	923	89.4 (87.4; 91.2)	89.6 (86.9; 91.8)
No	74	57	91.9 (81.7; 96.7)	90.1 (nd)
Condom use at last sex with non-paying partner				
Yes	172	142	90.4 (84.7; 94.2)	90.9 (83.3; 95.2)
No	384	316	87.8 (84.0; 90.8)	87.0 (82.3; 90.6)
History of previous incarceration in last 12 month				
Yes	13	10	76.9 (42.8; 93.7)	51.3 (nd)
No	1,321	1,072	89.7 (87.8; 91.3)	90.1 (87.6; 92.0)
Current alcohol consumption				
Yes	819	672	89.6 (87.2; 91.6)	88.3 (85.0; 91.0)
No	519	410	89.3 (86.1; 91.8)	91.4 (87.4; 94.2)
Lifetime history of drug use				
Yes	101	85	91.4 (83.5; 95.7)	90.8 (nd)
No	1,237	997	89.3 (87.4; 91.0)	89.4 (86.8; 91.5)
HIV knowledge				
Sufficient	303	277	91.4 (87.7; 94.1)	90.3 (84.5; 94.0)
Insufficient	1,035	805	88.9 (86.6; 90.7)	89.3 (86.4; 91.6)
Number of biological children				
Neither	441	344	87.5 (83.9; 90.5)	88.8 (84.2; 92.2)
One child	421	332	89.2 (85.7; 92.0)	89.1 (84.0; 92.7)
Two or three	384	324	90.5 (87.0; 93.1)	89.2 (84.3; 92.8)
More than three	92	82	95.3 (88.0; 98.3)	98.2 (94.6; 99.4)
Membership in an association				
Yes	104	91	93.8 (86.7; 97.2)	89.2 (73.9; 96.0)
No	1,234	991	89.1 (87.1; 90.8)	89.5 (87.0; 91.6)

### 3.2 Willingness to use HIV self-testing

The rates of willingness to use HIVST among FSWs are presented in Table 1. Among the 1,338 FSWs, 1,082 agreed to use HIVST, for a rate of 89.5% (95% CI: [87.0; 91.6]). The proportion of FSWs who were willing to use HIVST for different subgroups of demographics and risk behaviors was high, ranging from 85.1 to 98.2%. This proportion was highest (98.2%) among FSWs who had more than three children and lowest (51.3%) among FSWs who had a history of previous incarceration in the last 12 months before the data collection. The proportion of FSWs who were willing to use the HIVST rate appeared to be affected by monthly income, but from an income of 700$ to 5,014$, the prevalence was the lowest at 85.8%.

### 3.3 Factors associated with FSW willingness to use HIV self-testing

Table 2 below displays the results of the modified Poisson regression on the willingness to use HIVST among FSWs in Burkina Faso. As reported in the table, the proportion of willingness to use HIVST was 10% greater among FSWs who were married than among those who were not married (single), with a significant *p*-value (*p* = 0.034). The proportion of willingness to use HIVST among FSWs whose first sex was less than 18 years (<18 years) was 14% higher than that among those whose first sex was at least 18 years old (*p* = 0.024). The proportion was significantly (*p* = 0.010) higher among those who used condoms during their last sex with a non-paying partner. Compared with those who have a current alcohol consumption, female sex workers who did not have a current alcohol consumption had a 6% higher willingness rate to use HIVST, with a significant p-value (*p* = 0.026). Those who are members of an association of FSWs have a 10% higher rate of willingness to use HIVST than those who are not members of any association of FSWs (*p* = 0.014).

**Table 2:** Factors associated with willingness to use HIV self-testing among FSWs.

Variable	Bivariable analysis		Multivariable analysis	
Overall	Crude PR (95% CI)	*p*-value	Adjusted PR (95% CI)	*p*-value
Age				
<25				
> = 25	1.01 (0.97; 1.06)	0.560	1.00 (0.91; 1.04)	0.997
Current marital status				
Single				
Married	1.04 (0.91; 1.19)	0.538	1.10 (1.01; 1.20)	0.034
Divorced or widowed	0.97 (0.92; 1.02)	0.196	1.04 (0.93; 1.15)	0.513
Educational level				
Illiterate				
Primary school	0.96 (0.89; 1.04)	0.351	1.00 (0.85; 1.20)	0.985
Secondary or high school	1.03 (0.96; 1.11)	0.427	1.09 (0.93; 1.27)	0.277
Monthly income				
14$–100$				
100$–250$	1.03 (0.93; 1.15)	0.556	1.14 (0.88; 1.49)	0.320
250$–700$	1.04 (0.94; 1.16)	0.427	1.18 (0.88; 1.57)	0.269
700$–5014$	0.99 (0.86; 1.13)	0.849	1.00 (0.76; 1.33)	0.972
Age at first sex				
<18	1.03 (0.96; 1.11)	0.418	1.14 (1.02; 1.29)	0.024
> = 18				
Age at initiation of sex work				
<18				
> = 18	1.06 (0.97; 1.17)	0.192	1.06 (0.98; 1.14)	0.138
History of injection drug use (ever)				
Yes				
No	0.90 (0.87; 0.92)	<0.001	1.03 (0.89; 1.18)	0.734
Condom use at last sex with paying partner				
Yes				
No	1.01 (0.91; 1.11)	0.886	1.02 (0.89; 1.17)	0.770
Condom use at last sex with non-paying partner				
Yes	1.04 (0.98; 1.10)	0.155	1.07 (1.02; 1.13)	0.010
No				
History of previous incarceration in last 12 month				
Yes				
No	1.76 (0.85; 3.64)	0.129	2.30 (0.78; 6.77)	0.129
Current alcohol consumption				
Yes				
No	1.03 (1.00; 1.07)	0.057	1.06 (1.01; 1.12)	0.026
Lifetime history of drug use				
Yes				
No	0.98 (0.92; 1.05)	0.604	0.90 (0.84; 0.97)	0.004
HIV knowledge				
Suffisante				
Insufficient	0.99 (0.95; 1.03)	0.604	1.02 (0.91; 1.15)	0.716
Number of biological children				
Neither				
One child	1.00 (0.95; 1.06)	0.891	1.04 (0.96; 1.12)	0.334
Two or three	1.01 (0.96; 1.06)	0.823	1.02 (0.95; 1.11)	0.539
More than three	1.11 (1.06; 1.16)	<0.001	1.13 (0.99; 1.29)	0.060
Member of an association				
Yes	1.00 (0.87; 1.14)	0.971	1.10 (1.02; 1.18)	0.014
No				

## 4 Discussion

### 4.1 Key findings

This is the first analysis of HIVST among FSWs in Burkina Faso. Our findings suggest a high rate (89.5%) of willingness to use HIVST among FSWs in Burkina Faso. The willingness to use HIVST seems to be greater among FSWs who are married, those who initiated sex before 18 years of age, those who did not use condoms at last sex with non-paying partners, those who used drugs, those of did not consume alcohol, and those who are member of FSW association.

### 4.2 FSW willingness to use HIV self-testing

Compared to other studies, our study revealed a high rate of willingness to use HIVST among FSWs (89.5%) in Burkina Faso. Kim et al. (17) reported a lower rate (30.1%) of willingness to use HIVST among FSWs in Malaysia. While a qualitative study conducted in western Africa (Mali, Cote d’Ivoire, and Senegal) showed positive attitudes and willingness of FSW toward HIVST use (18), studies that focused on the willingness to use HIVST are sparse. However, many studies have assessed the acceptability and uptake of HIVST among key populations in SSA. Figueroa et al. (19) reported in their literature review that three of 23 studies on HIVST focused on FSW. Witzel et al. (20) noted that among 11 RCTs on HIVST compared with standard HIV testing, only three RCTs focused on HIVST among FSWs. The HIVST acceptability rate appears to be high in sub-Saharan Africa. Indeed, the HIVST rate reported in studies is close to 59.3% in Ethiopia (21). A systematic review revealed greater acceptability of HIVST among FSWs (19, 20). In particular, oral HIVST has a high acceptability rate, close to 90.5% in Morocco (22) and 81% in Botswana (23). Among other populations, studies have reported various levels of HIVST acceptance rates. In 2020, 70.4% of university students in Nigeria (24) and 94.3% in Senegal were first-time testers (25), and 84.5% were among the general population in Zimbabwe, 76.5% in Malawi, and 64.5% in South Africa. The STAR initiative in 13 countries has reported good acceptance of HIVST; the majority of participants found the test kits easy to use and interpret, and approximately two-thirds were willing to pay for HIVST (26). However, studies have shown some limits regarding the use of HIVST. The main limitation was the linkage HIV care service including confirmation test and ART initiation. Kra et al. (27, 28) reported that only 56% of the key population with HIVST positive was linked to a confirmation test. In 2021, Neuman et al. reported a limited effect of HIVST on ART initiation compared with that of standard testing services alone (29). From the ATLAS study, we learned that FSWs were reluctant to promote HIVST use among their casual clients (18). However, in another context, a recent community-based study showed a good linkage between confirmatory testing (89%) and ART initiation (85%), and 69% of participants were willing to pay for the HIVST kit (26). In Burkina Faso, a national strategy plan was drafted in 2022 to improve the application of a differentiated service approach known to significantly improve HIV cascades of care (30–32). From this plan, we noted that FSWs are among the main population for whom this approach is implemented. The approach included HIV testing, index testing, and HIVST as new strategies for HIV testing in addition to community facility-based HIV testing. Our findings suggest a good framework for scaling up HIVST services among FSWs in Burkina Faso. This approach might also accelerate the achievement of the first 95 since studies have shown that peer-assisted HIVST increases the number of newly diagnosed HIV-positive cases (33).

### 4.3 Determinant of FSW willingness to use HIVST

According to studies, various factors increase the likelihood of FSW’s willingness to use HIVST, depending on the setting. In our study, the age of initiation of sex work was not associated with FSW’s willingness to use HIVST. However, Nibret Eskezia (21) noted that spending more than 5 years of sex work increased the likelihood of HIVST acceptability. Age at first sexual intercourse was associated with HIVST. Nibret Eskezia et al. noted that FSWs aged >19 years at first sexual debut are 3.23 times more likely to accept HIVST. In our study, we did not assess knowledge about HIVST. Studies have shown good acceptability of HIVST among FSWs with good knowledge of HIVST (21). The association between risky sexual behavior and HIV testing is often difficult to interpret because of important desirability bias even if the data collector is well trained to avoid it. Indeed, our study reported that risky sexual behavior, such as not using a condom, was associated with a lower rate of FSW willingness to use HIVST. However, Stalter et al. noted that individuals who reported high-risk behaviors were more likely to repeat HIV tests (34). Manu et al. reported no significant association between risky sexual behaviors and HIV testing (35).

### 4.4 Public health implications

Studies have reported low rates of HIV testing among FSWs (36–38). Our previous study showed that only half (57.8%) of FSWs reported having been tested for HIV in the last 12 months suggesting an important gap in HIV testing in this population (8). There are many barriers to the use of standard HIV testing services. Among these barriers are stigma and discrimination regarding HIV-positive individuals and sex work (38). Many studies have highlighted that HIVST may address this issue, as it is safe, confidential, and increases the acceptability and frequency of HIV testing (20). Indeed, HIVST increased testing uptake by 36% compared with standard HIV testing among FSWs (20). A modeling study from the ATLAS project revealed that HIVST might increase the HIV diagnosis rate by 1.3% in Côte d’Ivoire, 10.6% in Senegal, and 3.6% in Mali by the end of 2028 (39). HIVST is projected to reduce disparities in access to HIV testing among key populations and their partners in Western Africa (39). Regarding the benefit of HIVST in HIV prevention, we recommended scaling up this intervention in Burkina Faso. This might help fill the gap in HIV testing among FSWs in the country. However, the linkage to HIV care seems to be low compared with that to standard care (20). Indeed, the systematic review revealed a lower linkage to HIV care via HIVST than to standard HIV care (20). Linkage to care seems to be the key aspect of HIVST, which needs to be addressed to improve the benefit of HIVST. Indeed, Kouassi et al. noted that only 56% of participants with a positive HIVST result followed up with confirmatory HIV tests. While compared to standard HIV testing, HIVST seems to better screen HIV-positive FSWs (33). This approach might help reach FSWs who are not able to use facility-based HIV testing services. The ATLAS HIVST strategy revealed that HIVST increased HIV testing coverage by reaching the underserved population across West Africa (40). Since studies such as that of Mavhu et al. (45) noted that HIVST is preferred over health provider-based HIV testing, public health policymakers in Burkina Faso must promote HIVST among FSWs to increase the HIV testing rate and reach the WHO recommendation regarding HIV testing among FSWs (7). The program level cost of HIVST strategies seems to be higher than HIV standard service (41); however, a part of the cost may be supported by the population since they are willing to pay for HIVST kit (26). Promoting HIVST needs to be combined with risk behavior control intervention among FSWs since evidence among MSM shows that risk behavior seems to increase after HIVST introduction (42). It is also necessary to take into account strategies that might support FSWs who tested HIV positive to disclose their status to their partners and invite them for testing (43).

### 4.5 Strengths and limitations

Face-to-face interviews in the context of sex work stigma might cause social desirability. There is recall bias regarding some characteristics reported as independent variables in this study. The study was conducted in five cities of the country, and the results might not be generalizable to all FSWs in the country. Some factors, such as self-perceived risk known to be associated with HIV testing, were not collected. However, this is the first quantitative assessment of HIVST acceptability among FSWs in Burkina Faso. We also used the RDS approach, which is among the best approaches for studying hidden populations (11, 44).

## 5 Conclusion

This study revealed a high HIVST willingness rate among FSWs in Burkina Faso. This rate seems to be higher among FSWs who were members of peer group associations suggesting that peer approach might be used to improve HIV testing. In the context of the ongoing implementation of self-testing as a new approach to increased HIV testing coverage, particularly among FSWs, our findings suggest that this strategy is favorable. As studies have shown, WHO recommendations regarding HIV testing frequency among FSW intensive efforts are still needed to improve the HIV testing rate.

## Data Availability

The raw data supporting the conclusions of this article will be made available by the authors, without undue reservation.
